# Boundary-associated propagation of a processed pseudogene dissects pre-existing limitations of genome annotation in the T2T era

**DOI:** 10.1186/s13100-026-00394-z

**Published:** 2026-02-17

**Authors:** Min-Gyu Lee

**Affiliations:** School of Life Sciences and Biotechnology, Korea University, Anam-dong, Sungbuk-gu, Seoul, Republic of Korea

**Keywords:** Processed pseudogene, Segmental duplication, Chromosomal boundary, Retrotransposition, Centromeric duplication, SEPTIN14, Annotation error, Retrogene, T2T unique, T2T annotation

## Abstract

**Background:**

Processed pseudogenes and retrogenes are defined by their RNA-mediated origin and, by virtue of this origin-based definition, are often interpreted as discrete genomic insertions. The completion of telomere-to-telomere (T2T) reference assemblies has substantially improved the resolution of segmental duplication architectures and centromeric satellite sequences that were previously inaccessible, allowing genomic structural contexts that were effectively invisible in earlier references to be directly examined.

**Results:**

Using the SEPTIN14P-CICP locus family as a case study, chain-based comparative analyses showed that a genomic window spanning the SEPTIN14 3′ terminal exon and the adjacent processed pseudogene CICP12 is dispersed into multiple segmental duplication-associated units across great apes, rather than being maintained as a single orthologous locus. Genome-wide analyses further indicated that annotated CICP loci preferentially localize within segmental duplication blocks and accumulate near pericentromeric or subtelomeric regions. Despite this duplication-associated dispersion, codon-based selection analyses revealed pervasive purifying selection acting on the full-length SEPTIN14 coding sequence and its 3′ terminal exon, arguing against a model in which the terminal exon was newly formed through segmental duplication. Together, these results show that when highly conserved, strongly constrained coding regions are embedded within segmental duplication-rich regions, co-dispersed processed pseudogene copies can be interpreted as distinct from independently generated LINE-1-mediated insertions and as reflecting secondary structural propagation.

**Conclusions:**

When considered in light of origin-based definitions of processed pseudogenes and retrogenes, and specifically within duplication-rich and structurally unstable genomic regions resolved by T2T-level assemblies, these results suggest that multiple annotated loci can arise through secondary propagation of a single RNA-derived insertion. Under such contexts, incorporation of selective constraint and cross-species conservation enables more reliable distinction between source insertions and their secondarily propagated copies. This case study highlights a limitation of current annotation frameworks and demonstrates the need for more precise annotation that incorporates evolutionary and structural context in the T2T era.

**Supplementary Information:**

The online version contains supplementary material available at 10.1186/s13100-026-00394-z.

## Background

 Processed pseudogenes (also termed retrogenes or retrocopies in origin-based usage) are classically defined as intronless gene copies generated by RNA-mediated retrotransposition, typically derived from spliced mRNA and integrated back into the genome as cDNA-like inserts [1, 2]. In many cases, such copies lack canonical regulatory context at the insertion site and, when nonfunctional, accumulate disabling mutations consistent with neutral sequence decay over evolutionary time [1, 2]. Since early descriptions, processed pseudogenes have been widely cataloged in mammalian genomes and used as historical readouts of retrotransposition activity, often in the context of LINE-1–mediated mobilization [3, 4].

With the accumulation of large-scale genome annotations, however, it has become increasingly clear that processed pseudogene annotations are frequently embedded within duplication-rich genomic contexts. Segmental duplications (SDs) are enriched in pericentromeric and subtelomeric regions and are a major source of assembly and annotation complexity [5–7]. Because SD-rich regions undergo frequent structural rearrangement and copy-number variation, the present-day genomic distribution of retroposed sequence annotations can reflect not only the initial RNA-mediated origin but also subsequent large-scale structural propagation [8, 9].

A persistent conceptual ambiguity in processed pseudogene annotation arises when “origin” and “propagation” are implicitly conflated. While the term “processed pseudogene” is intended to describe an RNA-mediated origin, annotation catalogs may be interpreted as though each annotated locus represents an independent retrotransposition event. In duplication-rich regions, a single retroposed insertion can be secondarily copied, fragmented, or redistributed through SD-mediated duplication and rearrangement, generating multiple annotation instances that share a common origin but differ in genomic location and local structure [10].

This ambiguity has been exacerbated by historical limitations of reference genome assemblies in highly repetitive regions. SD boundaries and pericentromeric sequences have been difficult to resolve and are often underrepresented or misrepresented in incomplete assemblies and short-read–limited frameworks, complicating both structural interpretation and gene copy annotation [11–13]. In such contexts, pseudogene-rich loci may be missing, partially represented, or annotated with inconsistent boundaries across references. Consistent with this, current genome annotation pipelines are known to exhibit reduced reproducibility in repetitive sequence contexts, including transposable elements and duplication-rich regions, highlighting intrinsic limitations in annotation confidence in such genomic environments [14].

The completion of telomere-to-telomere (T2T) human genome assemblies has fundamentally altered this landscape. The T2T-CHM13 assembly resolved many previously inaccessible regions, including centromeric satellites and substantial SD content, enabling direct re-examination of gene copy annotations in these contexts [15, 16]. Importantly, complete references make it more feasible to assess whether clusters of processed pseudogene annotations reflect multiple independent insertions or instead secondary structural propagation of a single ancestral insertion within SD-rich architecture [17].

In parallel, comparative genomics across primate lineages has revealed extensive lineage-specific structural variation, particularly involving duplication and copy-number changes in SD-rich regions [9, 18]. Such variation complicates interpretation of pseudogene copy number and distribution because orthology relationships can be obscured by fragmentation and dispersal of homologous sequence blocks across multiple chromosomes. Chain- and net-based alignment frameworks provide a way to trace these relationships without presupposing one-to-one locus correspondence, enabling structural redistribution to be analyzed separately from claims about insertion timing or locus “identity” [19].

In this study, the SEPTIN14P–CICP locus family is used as a focused case to dissect the distinction between processed pseudogene origin and subsequent structural propagation. By integrating T2T-resolved human assemblies, chain-based comparative analyses across great apes, and selection-pressure analyses on coding regions, this work aims to (i) characterize how SD-associated genomic contexts influence processed pseudogene annotation, (ii) identify structural failure modes that inflate apparent pseudogene copy number, and (iii) test whether SD-mediated dispersion is compatible with de novo exon formation or instead reflects redistribution of pre-existing coding elements.

Together, these analyses provide a framework for interpreting processed pseudogene annotations in duplication-rich genomic regions under T2T-scale assemblies, emphasizing the need to conceptually separate insertion origin from post-insertion propagation when inferring evolutionary histories from origin-based gene copy annotations.

In this study, the term boundary-associated refers to genomic regions encompassing or adjacent to telomeric and centromeric sequences, including loci that remained unresolved or represented as assembly gaps prior to T2T-level human genome assemblies. These regions correspond to structurally complex genomic environments that were historically inaccessible to sequence-based annotation and comparative analysis. Importantly, the use of boundary-associated here denotes positional and assembly-related context, rather than implying a mechanistic role in duplication initiation or insertion origin.

## Result

**Fig. 1:**
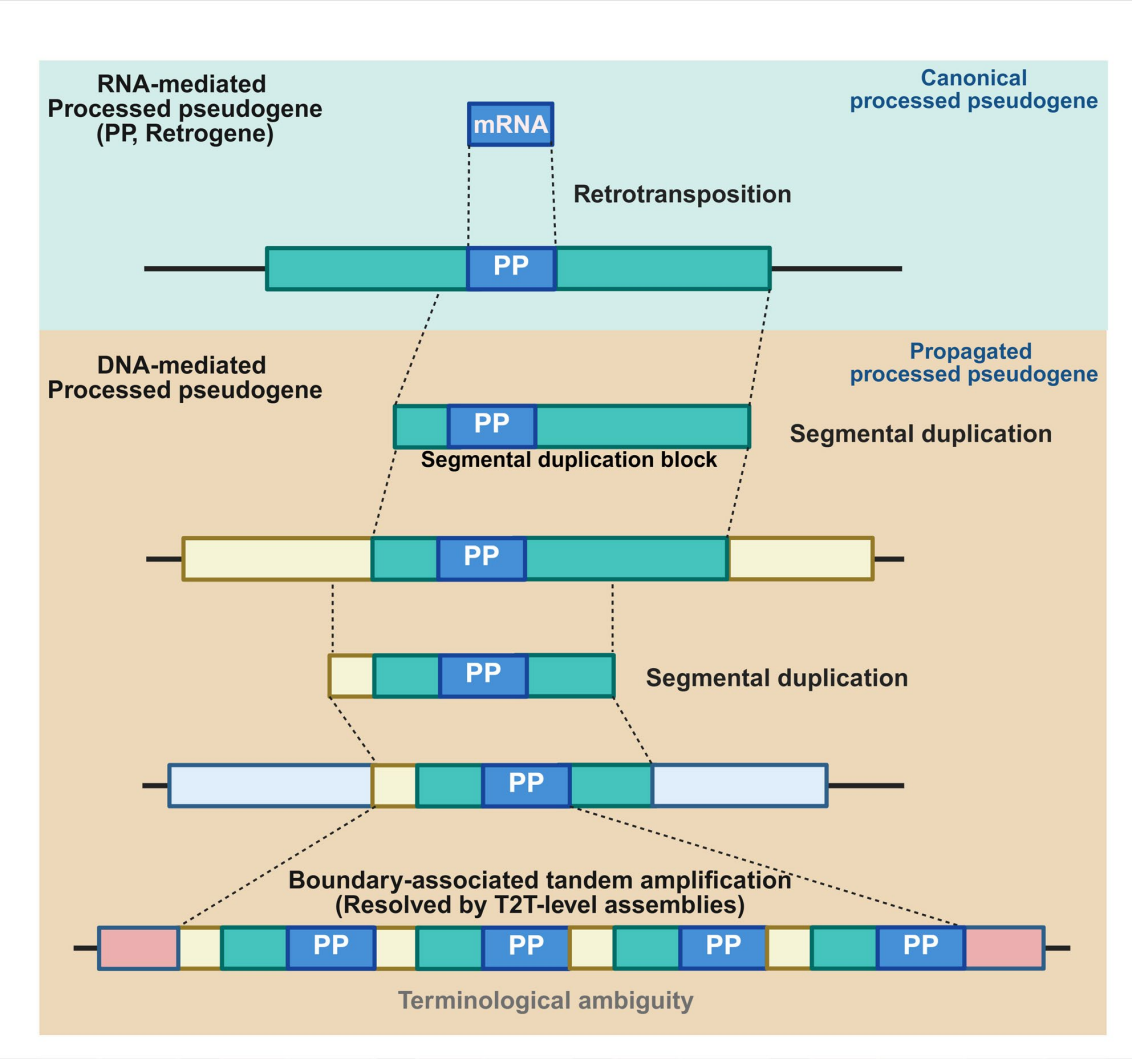
Conceptual separation of origin and structural propagation in processed pseudogenes. Schematic illustration distinguishing the origin of processed pseudogenes from their subsequent structural propagation.The upper panel depicts a canonical RNA-mediated processed pseudogene (PP, retrocopy) generated by LINE-1-mediated retrotransposition of an mRNA transcript. This class represents the classical definition of processed pseudogenes based on their origin. The lower panels illustrate scenarios in which an initially RNA-mediated processed pseudogene becomes embedded within segmental duplication blocks and undergoes DNA-mediated propagation, including boundary-associated tandem amplification resolved only by T2T-level assemblies.These structurally propagated copies retain their processed pseudogene origin but introduce terminological ambiguity when classified solely by sequence structure. This framework highlights the necessity of separating origin-based definitions from context-dependent structural outcomes in processed pseudogene annotation

An overview of the conceptual framework used in this study is shown in Figure 1.

### Segmental duplication units containing the CICP12 insertion

**Fig. 2:**
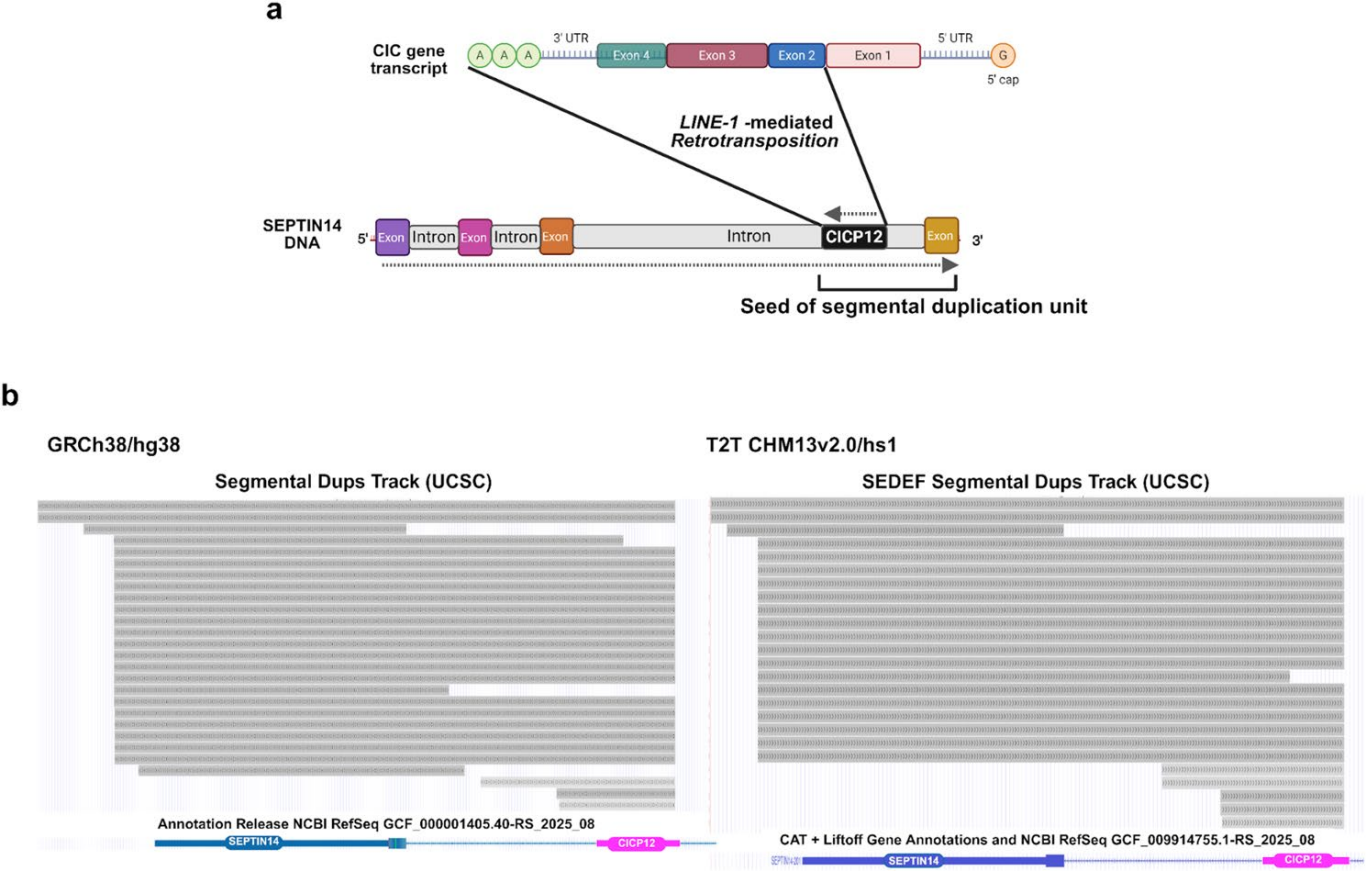
Genomic context of the SEPTIN14-CICP12 locus and associated segmental duplication tracks. **a** Schematic representation of a CIC gene transcript and its genomic relationship to the SEPTIN14 locus, showing the position of CICP12 within an intronic region near the 3′ end of SEPTIN14. **b** Comparison of segmental duplication tracks surrounding the SEPTIN14-CICP12 region in GRCh38/hg38 and T2T CHM13v2.0/hs1, displayed using UCSC Segmental Dups (hg38) and SEDEF-based segmental duplication tracks (T2T). Corresponding gene annotation tracks are shown below for each assembly

The SEPTIN14-CICP12 locus shown in Fig. 2 comprises a composite configuration in which CICP12 is inserted within an intronic region near the 3′ end of SEPTIN14, with the insertion inferred to have occurred on the opposite strand. In both the GRCh38/hg38 and T2T CHM13v2.0/hs1 assemblies, the CICP12 insertion site is fully contained within continuous segmental duplication units spanning from one duplication boundary to the other.

In current RefSeq- and Ensembl-based annotations, most SEPTIN14-derived paralogous loci associated with this region (SEPTIN14P family members) are classified as processed pseudogenes, reflecting their intronless structure and sequence similarity to the parental SEPTIN14 transcript, consistent with the observation that only the 3′ terminal exon of SEPTIN14 is represented in these propagated copies.

Tissue-level expression concordance was quantified for one propagated pair derived from this locus, CICP16 and SEPTIN14P4, using GTEx tissue-wise median expression values (Fig. S2). Across 54 tissues, strong correlations were observed (Spearman ρ = 0.983; Pearson *r* = 0.979), with a linear regression slope of 1.21. Leave-one-tissue-out analysis yielded a minimum Spearman correlation of 0.982 (Fig. S2a). This pattern is not observed across most propagated pairs and therefore does not represent a necessary condition, but may constitute a sufficient condition in this specific configuration. A representative expression analysis for one CICP-SEPTIN14P pair is described in the Supplementary Methods.

Similar annotation behavior is also observed at additional loci embedded within protein-coding gene contexts, where sequences annotated as processed pseudogenes are distributed across segmental duplication blocks (Fig. S6). These examples illustrate that ambiguity in origin inference is not unique to the SEPTIN14-CICP locus but can arise more broadly in duplication-rich genomic regions.

### Genome-wide distribution of CICP loci across GRCh38 and T2T assemblies

**Fig. 3:**
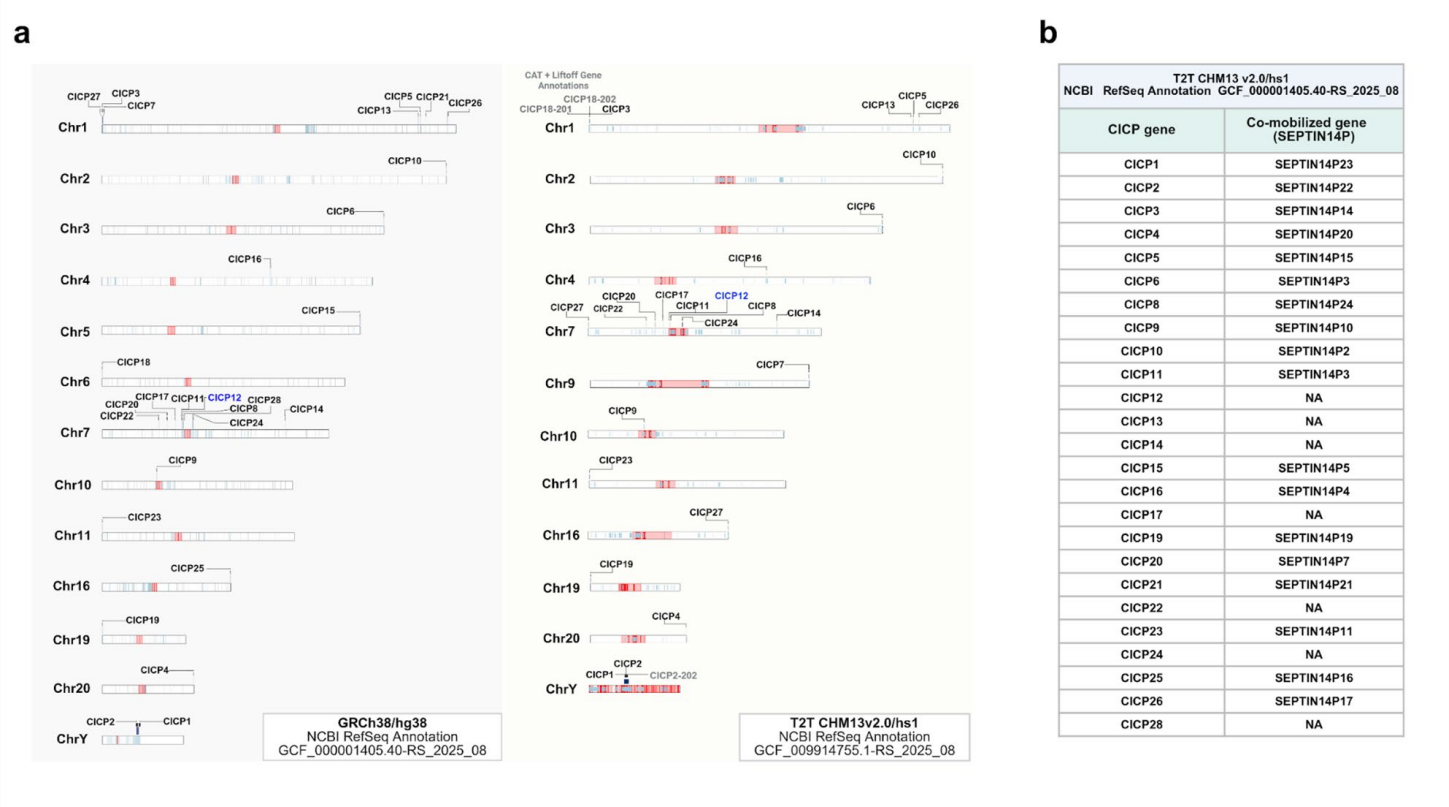
Genome-wide distribution of CICP loci and centromere-associated duplication contexts in GRCh38 and T2T assemblies. **a** Chromosomal distribution of CICP loci across the human genome in GRCh38/hg38 and T2T CHM13v2.0/hs1 based on NCBI RefSeq annotations. Blue blocks represent segmental duplication (SD) regions in each assembly. Red-highlighted regions indicate centromere-associated sequences, corresponding to unresolved centromeric gaps in GRCh38 and explicitly annotated centromeric satellite sequences in the T2T assembly. The positions of individual CICP loci are shown along each chromosome to facilitate direct comparison between assemblies. **b** Summary table of CICP loci annotated in the T2T CHM13v2.0/hs1 assembly, listing corresponding co-mobilized SEPTIN14 pseudogene annotations where present. Loci without an associated SEPTIN14P annotation are indicated as NA

The genome-wide chromosomal distribution of CICP loci was examined using NCBI RefSeq annotations in the GRCh38/hg38 and T2T CHM13v2.0/hs1 assemblies (Fig. 3a). In this representation, light blue blocks denote annotated segmental duplication (SD) regions in each assembly, while red-highlighted regions in the T2T assembly indicate annotated centromeric satellite sequences.

Across both assemblies, all annotated CICP genes were located within SD blocks, as indicated by their consistent overlap with light blue regions along the chromosomes. The locus corresponding to the inferred original CICP12 was positioned within a centromeric satellite region in the T2T assembly, whereas in the GRCh38 assembly the corresponding locus was observed to be adjacent to the centromeric region.

CICP loci were not evenly distributed across the genome but showed a pronounced clustering on chromosome 7, where multiple CICP copies were observed in close proximity. In addition, the majority of CICP loci were positioned toward subtelomeric or pericentromeric regions, consistent with an intrinsic, non-uniform chromosomal distribution pattern. The chromosomal positions of individual CICP loci in each assembly are shown to facilitate direct comparison between GRCh38 and T2T, and assembly-dependent genomic coordinates for all identified loci are provided in Fig. S1.

### Lineage-specific structural dispersion of the SEPTIN14-CICP12 genomic window across great apes

Comparative synteny analysis indicates clear differences in evolutionary stability between the parental CIC gene and SEPTIN14. CIC is consistently detected within a conserved syntenic block across placental mammals, indicating long-term stability of its genomic context. In contrast, SEPTIN14 shows evidence of later stabilization, with syntenic conservation primarily observed within primates, consistent with a more restricted evolutionary persistence relative to CIC (Fig. S3a). This primate-centered conservation is accompanied by increased structural variability of the SEPTIN14-associated genomic region.

Multi-species alignments further show that continuity of the SEPTIN14-associated region becomes detectable from drill onward, whereas increasing discontinuities are observed in more distant primates, reflecting both biological divergence and methodological limitations of multiple alignment approaches (Fig. S3b). Because these patterns do not allow precise inference of insertion timing, no attempt was made to assign a temporal origin based on these data, and structural interpretation was conservatively restricted to great apes.

**Fig. 4:**
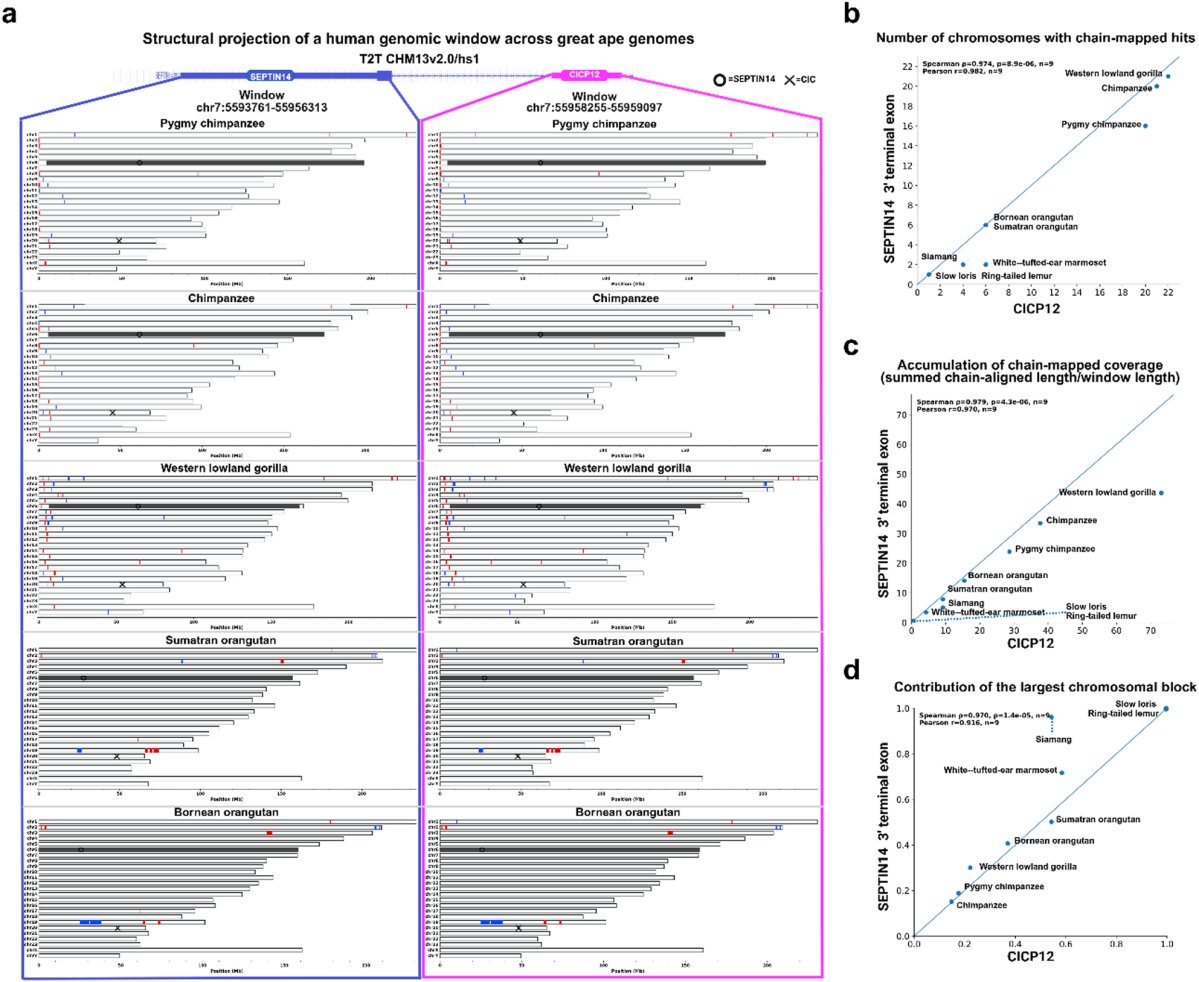
Chain-based projection of the SEPTIN14 3′ terminal exon and CICP12 locus across primate genomes. **a** Chain-based mapping of a human genomic window encompassing the SEPTIN14 3′ terminal exon (left, blue) and the adjacent CICP12 locus (right, magenta) from the T2T CHM13v2.0/hs1 assembly onto multiple primate genomes. For each species, horizontal bars represent individual chain-mapped alignments ordered by genomic position, illustrating how the same human window projects onto multiple chromosomes. Symbols indicate the projected positions of SEPTIN14 (circles) and CICP (crosses) within each alignment. **b** Scatter plot showing the number of distinct chromosomes with chain-mapped hits for the SEPTIN14 3′ terminal exon versus CICP12 across species. **c** Comparison of accumulated chain-mapped coverage, calculated as the sum of chain-aligned lengths normalized by the human window length, for the SEPTIN14 3′ terminal exon and CICP12. **d** Fractional contribution of the single largest chromosomal block to the total chain-mapped coverage for each locus, illustrating differences in how alignment coverage is distributed across chromosomes among species. All panels are based on chain-based structural projection and are intended to summarize alignment distribution patterns rather than infer functional or mechanistic relationships

Chain-based analyses indicate that the SEPTIN14-CICP12 genomic window does not remain as a single orthologous locus across great apes but is instead redistributed into multiple segmental duplication (SD) units in a lineage-specific manner (Fig. 4a; Fig. S3c). In all examined great ape genomes, chain-mapped segments corresponding to the same human genomic window were detected on multiple chromosomes, consistent with fragmentation of the window into discrete SD-associated blocks rather than linear conservation.

The extent of this fragmentation varies among great apes. Chimpanzee and pygmy chimpanzee exhibit an intermediate dispersion pattern, with chain-mapped segments distributed across several chromosomes, whereas western lowland gorilla shows a more pronounced dispersion, characterized by a larger number of chromosomes carrying smaller chain-mapped fragments (Fig. 4a, b). In contrast, orangutan genomes display fewer chromosomes with detectable projections, with a greater fraction of the window concentrated within larger chromosomal blocks, indicating reduced fragmentation relative to African great apes (Fig. 4a, b).

Quantitative comparisons further support concordant dispersion of the SEPTIN14 3′ terminal exon and CICP12 across great apes. The number of chromosomes containing chain-mapped hits for the two loci is strongly correlated across species, indicating that both loci participate in the same SD-associated redistribution process within each lineage (Fig. 4b). Accumulated chain-mapped coverage likewise shows concordant scaling between the two loci, reflecting shared patterns of coverage partitioning across chromosomes rather than locus-specific behavior (Fig. 4c).

Differences in the contribution of the largest chromosomal block further distinguish lineage-specific dispersion modes. In chimpanzee and western lowland gorilla, the largest block accounts for a smaller fraction of total coverage, consistent with increased fragmentation into multiple SD units, whereas orangutan shows a higher contribution from a single dominant block, indicating more limited dispersion (Fig. 4d).

### Structural dispersion of a T2T-unique genomic window at the SEPTIN14P-CICP segmental duplication boundary

**Fig. 5:**
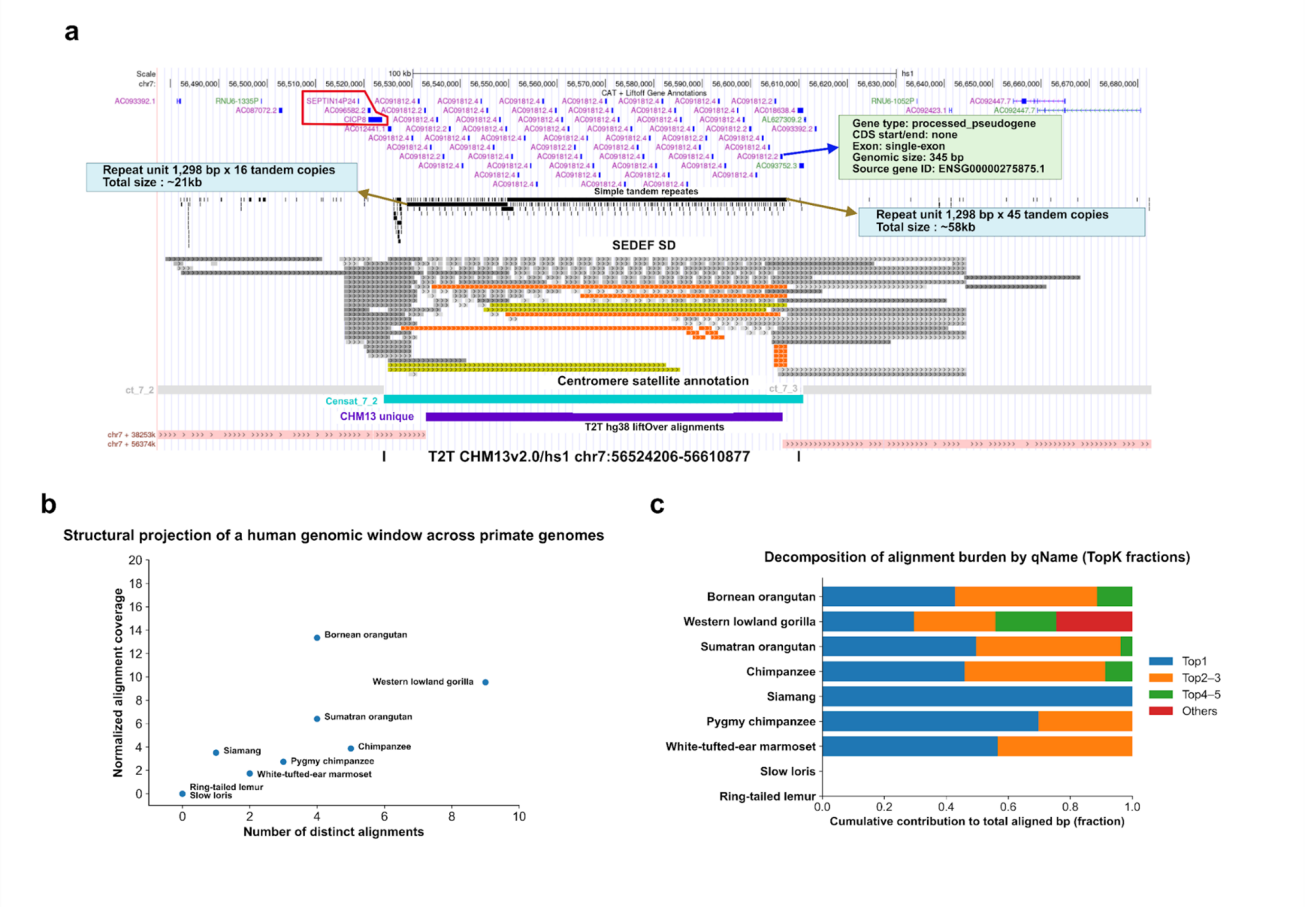
Structural organization and cross-species projection of a centromeric tandem repeat-associated genomic window in the T2T assembly. **a** Genome browser view of a repeat-rich region on chromosome 7 in the T2T CHM13v2.0/hs1 assembly, encompassing the SEPTIN14P24 locus and the nearby CICP8 processed pseudogene. The displayed window corresponds to the centromeric satellite annotation censat_7_2 (NovelTandem; chr7:56524206–56610877; genomic size 86,672 bp). Shown tracks include RefSeq and CAT+Liftoff gene annotations, simple tandem repeats, SEDEF-based segmental duplication blocks, centromeric satellite annotation, and T2T-hg38 liftover alignments, illustrating the local genomic context resolved by the T2T assembly. **b** Structural projection of the same censat_7_2 genomic window (chr7:56524206–56610877 in T2T CHM13v2.0/hs1) across multiple primate genomes, summarized by the number of distinct chain-based alignments and normalized alignment coverage for each species. Each point represents one species and reflects how this specific centromeric satellite window maps across primate assemblies. **c** Decomposition of the total chain-mapped alignment burden for the censat_7_2 window by qName, showing the cumulative contribution of the top-ranked alignment blocks (Top1, Top2-3, Top4-5, and others) to the total aligned base pairs in each species. This panel summarizes how alignment coverage of this defined centromeric satellite region is distributed across multiple alignment blocks rather than concentrated in a single block

The analyzed genomic interval corresponds to a centromeric satellite-associated region on chromosome 7 (censat_7_2) that is uniquely resolved in the T2T CHM13v2.0/hs1 assembly and was not accessible in GRCh38 due to unresolved centromeric or gap-associated sequences (Fig. 5a). This T2T-unique window (chr7:56524206–56610877) is located adjacent to the SEPTIN14P24 and CICP8 loci and overlaps the boundary of a SEPTIN14P-CICP-associated segmental duplication (SD) block. Within this interval, dense tandem repeats, extensive SEDEF-defined SD tracks, and explicit centromeric satellite annotation are observed, together with repeated annotation of closely related processed pseudogenes associated with the SEPTIN14P-CICP family (Fig. 5a).

Chain-based projection of this censat_7_2 window across primate genomes shows that homologous sequences do not map as a single contiguous orthologous locus but are distributed across multiple distinct genomic locations (Fig. 5b). Among great apes, orangutans exhibit higher normalized alignment coverage with fewer distinct chain-mapped targets, whereas chimpanzee and pygmy chimpanzee show intermediate dispersion. Western lowland gorilla displays the highest number of distinct alignments combined with lower normalized coverage, indicating increased fragmentation of homologous sequence across multiple SD-associated targets (Fig. 5b).

Decomposition of alignment burden by qName further highlights lineage-specific differences in dispersion (Fig. 5c). In orangutans, a larger fraction of aligned bases is contributed by a single dominant block, whereas African great apes show reduced dominance of the largest block and a greater contribution from multiple secondary alignments. Consistent with this pattern, aggregated alignment length distributions indicate longer contiguous segments in orangutans and shorter, more fragmented segments in chimpanzee and western lowland gorilla, reflecting increased fragmentation at the SD-block boundary in these lineages (Fig. S5).

### Segmental duplication-associated dispersion and conservation of the SEPTIN14 3′ terminal exon

Chain-based projection analyses were performed using a human genomic window spanning from the SEPTIN14 3′ terminal exon to the adjacent CICP12 locus. When this window was used as a query, homologous segments were not maintained as a single orthologous locus across primate genomes but were instead mapped to multiple chromosomal locations in a lineage-specific manner (Fig. S4). This pattern was particularly pronounced in great apes, in which the same window was repeatedly projected to multiple chromosomes, indicating structural dispersion associated with segmental duplication (SD) blocks rather than linear conservation.

The co-projection of the SEPTIN14 3′ terminal exon and CICP12 within the same dispersed blocks was consistently observed, supporting the interpretation that this window resides within an SD-associated genomic context that has undergone repeated structural rearrangements in great apes. These observations describe patterns of structural projection only and do not provide information on the timing or directionality of the underlying events.

Given the extensive SD-associated dispersion of the window containing the SEPTIN14 3′ terminal exon, it was next assessed whether this exon represents a coding element that was newly formed through SD in great apes or, alternatively, a pre-existing exon that has been structurally redistributed. To address this, codon-based selection analyses were performed on the SEPTIN14 coding sequence.

**Fig. 6:**
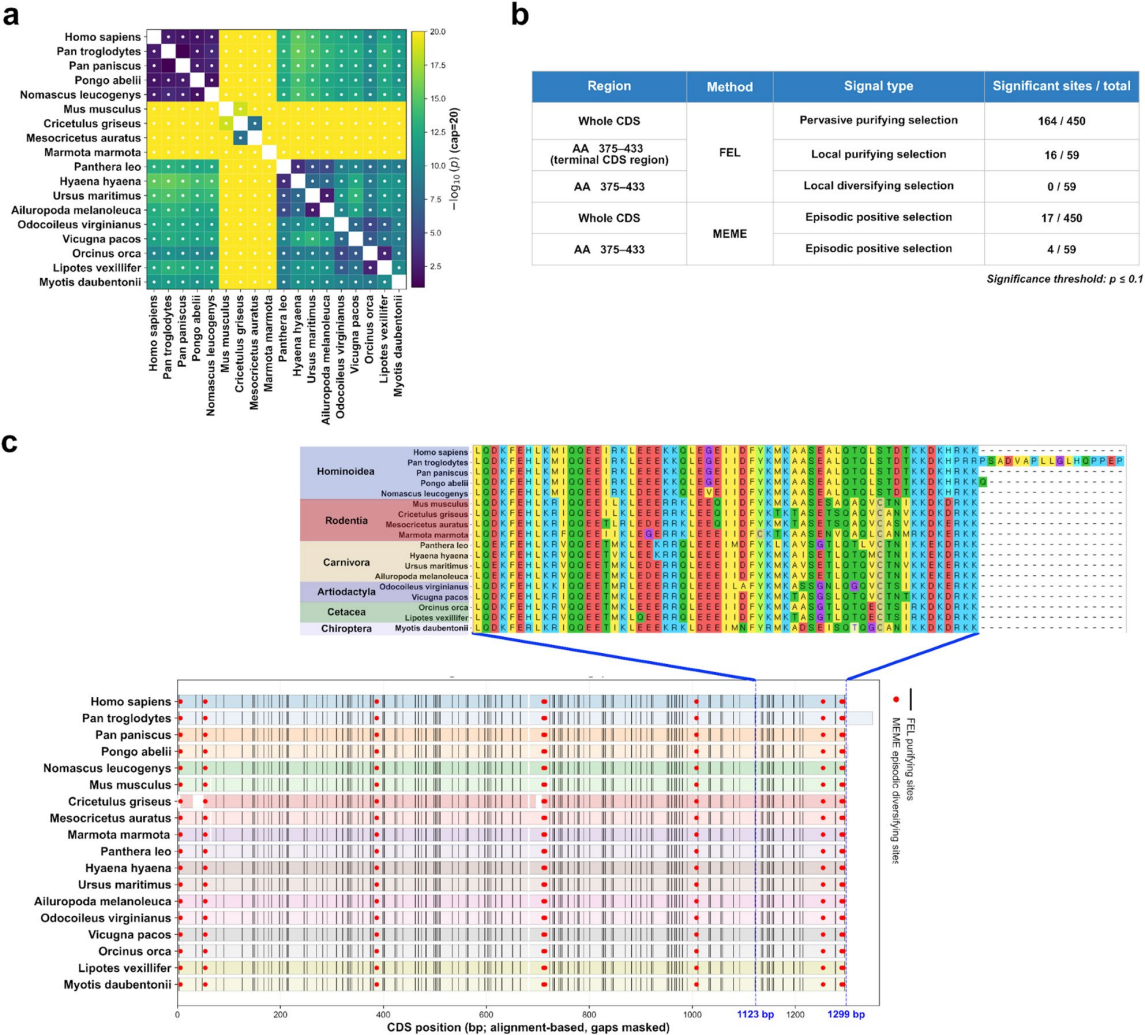
Selection and sequence conservation of the SEPTIN14 3′ terminal exon. **a** Heatmap summarizing pairwise codon-based comparisons across mammalian species for the SEPTIN14 coding sequence, with color intensity representing −log10(p) values and species arranged by phylogenetic grouping. **b** Summary table of site-based selection analyses using FEL and MEME applied to the full coding sequence and to the 3′ terminal exon region, reporting the dominant selection signal and the number of sites showing statistically significant evidence of selection at p ≤ 0.1. **c** Multiple sequence alignment of the SEPTIN14 coding sequence across representative mammals, showing amino acid conservation and site-wise mapping of inferred purifying and diversifying selection signals, with positions corresponding to the 3′ terminal exon indicated for comparison

Across mammalian lineages, the full-length SEPTIN14 coding sequence was found to be broadly conserved, with divergence patterns consistent with strong evolutionary constraint (Fig. 6a). Site-based selection analyses further showed that purifying selection was the dominant mode acting on SEPTIN14, with the majority of sites inferred to be under negative selection and little evidence for widespread positive selection (Fig. 6b).

When the analysis was restricted to the 3′ terminal exon, this region was likewise found to be predominantly under purifying selection. Although lineage-specific variation in selective pressure was observed, no systematic relaxation of constraint was detected, indicating that the terminal exon has remained evolutionarily conserved despite its localization within an SD-associated and structurally dynamic genomic window (Fig. 6b).

Taken together, these results do not support the hypothesis that the SEPTIN14 3′ terminal exon was newly formed through segmental duplication in great apes. Instead, the pervasive purifying selection acting on both the full-length coding sequence and the terminal exon indicates that this exon represents an evolutionarily conserved coding element that has been secondarily embedded within an SD-associated genomic context (Fig. 6c). 

## Discussion

This study uses the SEPTIN14–CICP locus family as a focused case to examine how origin-based gene copy annotations are interpreted when an RNA-derived insertion is situated within segmental duplication (SD)–associated genomic contexts. Rather than proposing a novel mechanism of pseudogene formation, the analyses are designed to evaluate how established annotation categories behave as reference genome completeness improves and duplication-rich regions become more consistently accessible in the telomere-to-telomere (T2T) era.

Across both the GRCh38 and T2T CHM13 assemblies, annotated CICP loci are consistently localized within SD blocks and display a non-uniform chromosomal distribution, with enrichment near pericentromeric and subtelomeric regions. In the T2T assembly, the inferred source CICP12 locus resides within a centromeric satellite-associated region, whereas corresponding loci in GRCh38 are positioned adjacent to unresolved centromeric gaps. The positional concordance observed between assemblies suggests that differences in locus representation primarily reflect reference completeness and assembly resolution, rather than multiple independent LINE-1-mediated retrotransposition events.

Chain-based comparative analyses further indicate that the genomic window encompassing the SEPTIN14 3′ terminal exon and the adjacent CICP12 sequence is distributed across multiple SD-associated units in great ape genomes, rather than being maintained as a single conserved orthologous locus. Notably, this dispersion pattern is observed for both the processed pseudogene and the adjacent coding exon, consistent with secondary propagation of an insertion-containing genomic unit through SDs. Differences in the extent and pattern of dispersion among great ape lineages are therefore more parsimoniously interpreted as lineage-specific behavior of duplication-rich genomic regions, rather than as locus-specific innovation or repeated retrotransposition events. For example, comparative analyses of great ape genomes have indicated that the gorilla lineage exhibits an especially high rate of segmental duplication relative to other ape species, consistent with lineage-specific enrichment of structurally dynamic regions [20].

The co-dispersion of the SEPTIN14 3′ terminal exon and CICP12 raises the possibility that the terminal exon itself might have originated via segmental duplication in great apes. However, codon-based selection analyses do not support this interpretation [21]. Instead, pervasive purifying selection across the full-length SEPTIN14 coding sequence, including its terminal exon, is consistent with long-term evolutionary constraint of a pre-existing coding element that has been secondarily embedded within a duplication-associated genomic context [22, 23]. This distinction underscores the importance of separating insertion origin from post-insertion structural propagation when interpreting exon evolution in duplication-rich regions.

More broadly, these observations suggest that RNA-derived insertions can become embedded within chromosomal regions that are structurally dynamic in a lineage-specific manner, including pericentromeric and subtelomeric environments. In such contexts, insertion-containing segments may be repeatedly incorporated into SD-associated units as part of larger structural rearrangements. Under this interpretation, the accumulation of processed pseudogene annotations in these regions does not imply a causal role in initiating duplication, but instead reflects the propensity of the surrounding genomic context to propagate existing sequence units. In this sense, processed pseudogene insertions may act as structural substrates within duplication-prone environments, becoming repeatedly represented as components of expanding SD blocks without acquiring functional relevance.

In duplication-rich regions where SDs encompass conserved sequence elements, it may therefore be possible to distinguish a LINE-1-mediated source insertion from its secondarily propagated copies by integrating sequence conservation, local synteny, and gene-level selective constraint, without implying functionality of the pseudogene itself. Processed pseudogenes and retrogenes are appropriately defined by their RNA-mediated origin, and this definition remains sufficient for describing the initial insertion event. However, existing origin-based classification frameworks are not designed to account for SD-driven post-insertion propagation that becomes apparent at T2T-level assembly resolution. As illustrated by the SEPTIN14–CICP locus, multiple annotated loci in duplication-rich regions may therefore reflect secondary propagation of a single insertion-containing genomic unit rather than repeated LINE-1-mediated retrotransposition events.

**Fig. 7:**
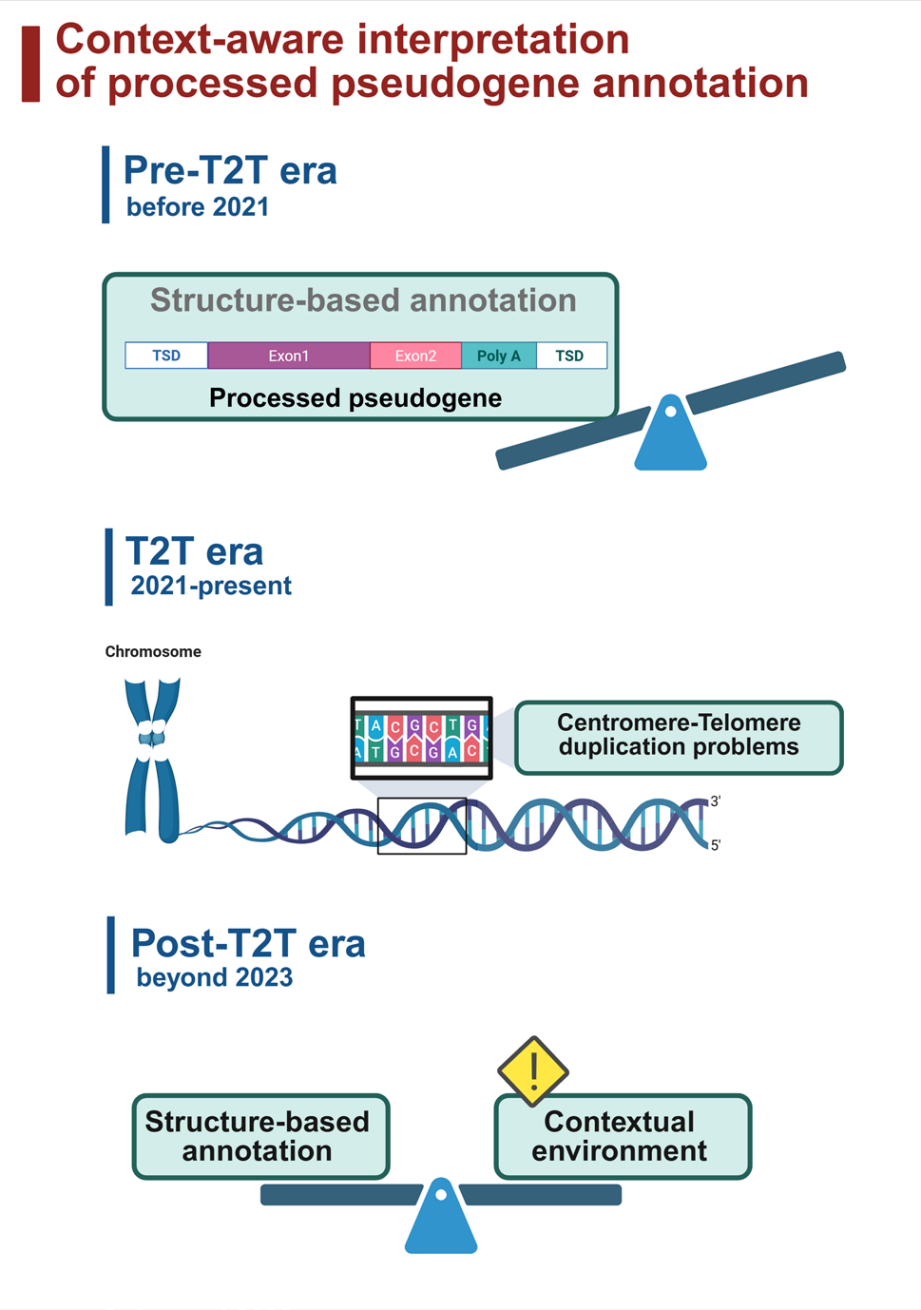
Limitations of structure-based processed pseudogene annotation revealed by T2T-level genomic context. This schematic summarizes how improvements in genome completeness from pre-T2T to post-T2T assemblies necessitate consideration of surrounding genomic context, particularly in duplication-rich boundary regions, when interpreting processed pseudogene annotations

This study is deliberately limited to a single locus family and does not seek to generalize these observations across all processed pseudogenes or retrogenes. Rather, the SEPTIN14-CICP locus provides a concrete case illustrating how long-standing annotation ambiguities can become more apparent as T2T-level reference assemblies are generated across species. As duplication-rich regions become increasingly accessible and better resolved, careful separation of insertion origin from post-insertion structural propagation will be important for the consistent interpretation of gene copy annotations, without necessitating changes to existing annotation frameworks (Fig. 7).

## Conclusion

Using the SEPTIN14-CICP locus family as a case study, this work shows that a single RNA-derived insertion can be represented as multiple annotated loci when embedded within segmental duplication-associated genomic contexts. In such cases, dispersion of processed pseudogene or retrogene annotations reflects secondary propagation of an insertion-containing unit rather than repeated LINE-1-mediated retrotransposition events. The evolutionary conservation of the SEPTIN14 coding sequence and its terminal exon further indicates that duplication-associated dispersion does not imply de novo exon formation.

These observations suggest that categories of processed pseudogene annotation that have long been regarded as challenging may warrant further consideration as T2T-level reference assemblies are increasingly being generated across species. As duplication-rich regions become more consistently accessible, careful separation of insertion origin from post-insertion propagation may become increasingly important, and existing definitions and annotation conventions may need to be revisited to maintain consistent interpretation across emerging high-completeness reference genomes.

## Methods

### Reference assemblies and gene annotation resources

Analyses were performed using the GRCh38/hg38 human reference genome [24] and the telomere-to-telomere human reference assembly T2T CHM13v2.0 (hs1) [15]. Gene annotations used for identification of CICP family members and SEPTIN14-derived loci were obtained from NCBI RefSeq for each assembly [25, 26], with Ensembl annotations inspected for cross-checking where necessary [25, 26]. For loci located in repeat-rich regions uniquely resolved in the T2T assembly, CAT+Liftoff annotations were additionally inspected to confirm local gene-model behavior [27, 28]. Segmental duplication (SD) intervals were obtained from the UCSC Segmental Dups track for GRCh38 [6] and from SEDEF-based SD annotations for the T2T assembly [17]. All genomic coordinates were handled in BED/TSV formats and processed using standard interval operations and custom scripts implemented in Python (v3.11).

### Centromeric satellite annotation and chromosome-level marking

Centromeric regions were defined using explicit centromeric satellite annotations. For the T2T CHM13v2.0 assembly, centromeric satellite intervals were obtained directly from the censat annotation tracks provided with the assembly [16]. For GRCh38, centromere-associated regions were inferred from annotated centromeric gaps and centromere boundary annotations [24]. These centromeric intervals were parsed as genomic coordinates and used to annotate chromosome-level visualizations, allowing explicit distinction between fully resolved centromeric satellite sequence in T2T and unresolved or gap-associated centromeric regions in GRCh38.

### Genome-wide identification and distribution of CICP loci

All loci annotated as members of the CICP family were extracted from NCBI RefSeq annotations for GRCh38/hg38 and T2T CHM13v2.0/hs1 [29]. Genomic coordinates were intersected with SD intervals to assess overlap with duplication blocks. Chromosomal distributions were summarized to evaluate non-uniform positioning along chromosomes and relative enrichment near pericentromeric or subtelomeric regions. Assembly-dependent differences were interpreted in the context of reference completeness rather than as independent biological events.

### Definition of genomic query windows

All locus-centered analyses were performed using genomic windows defined on the T2T CHM13 reference. These included windows spanning the SEPTIN14 3′ terminal exon, the adjacent CICP12 locus, and combined windows encompassing both elements, which are separated by 1,904 bp in the human reference genome. In addition, a repeat-rich T2T-unique window located at a SEPTIN14P-CICP-associated segmental duplication boundary (chr7:56524206–56610877) was selected for focused analysis. Window boundaries were fixed on the human reference and applied consistently across all comparative analyses.

### Primate genome assemblies

Comparative analyses were performed using publicly available primate genome assemblies. For each species, the primary (pri) assembly version was used as the target genome. Genome assembly information, including chromosome identifiers and chromosome lengths, was obtained from the corresponding NCBI Genome assembly pages [30]. Only assembled chromosomes represented by RefSeq accessions were used for downstream analyses. 

The following assemblies were used:

Chimpanzee (Pan troglodytes): GCA_028858775.2_NHGRI_mPanTro3-v2.0_pri.

Pygmy chimpanzee (Pan paniscus): GCA_029289425.2_NHGRI_mPanPan1-v2.0_pri.

Western lowland gorilla (Gorilla gorilla): GCA_029281585.2_NHGRI_mGorGor1-v2.0_pri.

Sumatran orangutan (Pongo abelii): GCA_028885655.2_NHGRI_mPonAbe1-v2.0_pri.

Bornean orangutan (Pongo pygmaeus): GCA_028885625.2_NHGRI_mPonPyg2-v2.0_pri.

Siamang (Symphalangus syndactylus): GCA_028878055.2_NHGRI_mSymSyn1-v2.0_pri.

White-tufted-ear marmoset (Callithrix jacchus): GCF_011100555.1_mCalJa1.2.pat.X.

Ring-tailed lemur (Lemur catta): GCF_020740605.2_mLemCat1.pri.

Slow loris (Nycticebus coucang): GCF_027406575.1_mNycCou1.pri.

### Chromosome size tables and qName mapping

For each target species, chromosome identifiers and chromosome lengths were extracted from NCBI Genome assembly data and compiled into species-specific chromosome size tables. These tables were used to scale chromosome representations in chromosome-level visualizations and to match chain alignment target identifiers (qNames) to assembled chromosomes. Chain alignment blocks whose qName did not correspond to assembled RefSeq chromosomes were excluded from chromosome-level summaries to ensure consistent chromosome-level interpretation.

### Chain alignment resources and chain-based projection

Whole-genome chain alignment files mapping the human reference to each target primate assembly were obtained from the UCSC Genome Browser database using the Table Browser interface [31, 32]. UCSC “all” chain files were used. Coordinate projection via liftover utilities was not applied. Human query windows defined on the reference genome were projected onto each target genome by parsing chain alignments and collecting all chain-mapped alignment blocks overlapping each query window, without enforcing one-to-one orthology [19].

### Quantification of accumulated chain-mapped coverage and dispersion

For each species and each human query window, accumulated chain-mapped coverage was calculated as the sum of chain-mapped alignment lengths normalized by the length of the human query window:

$$\text{Normalized accumulated chain-mapped coverage}=\sum L\_chain/ L\_window$$

where *L_chain* denotes the length of each chain-mapped alignment block and *L_window* denotes the length of the human query window [19]. Multi-mapping was allowed; thus, overlapping chain-mapped blocks were retained and their aligned lengths contributed additively to *Σ L_chain*. Structural dispersion was quantified independently as the number of distinct chain alignment targets (qNames) carrying at least one chain-mapped block for the given query window.

### Visualization and plotting

Chromosome-level visualizations were generated using species-specific chromosome size tables derived from NCBI assemblies. Chain-mapped alignment blocks corresponding to each query window were plotted as ticks or segments along scaled chromosome representations and ordered by genomic position. All custom plotting and figure generation were implemented in Python (v3.11) using matplotlib [33].

### Local genomic context inspection

For selected loci and genomic windows, genome browser views were inspected to qualitatively confirm annotation behavior and surrounding genomic features. Tracks examined included RefSeq and CAT+Liftoff gene models [25, 28, 34], segmental duplication intervals [6, 17], repeat annotations [35], and centromeric satellite annotations where applicable [16]. Comparisons between GRCh38 and T2T assemblies were restricted to descriptive differences attributable to assembly resolution and were used solely for contextual confirmation rather than for independent analytical inference. Local context inspection and track visualization were performed using the UCSC Genome Browser, with custom BED tracks uploaded as needed [31].

### Genome context analysis based on parental gene conservation

Genome browser-based analyses were performed to examine the local syntenic environment surrounding the parental genes CICP and SEPTIN14. Conservation of these parental genes was used as a positional anchor to assess the surrounding syntenic context, including neighboring gene arrangements and duplication-associated boundaries. This analysis was used to qualitatively examine the relative genomic environment and ordering relevant to locus origin. These observations were descriptive and were not used to infer functional conservation or to establish causal evolutionary relationships.

### Sequence collection and codon-aware alignment

Orthologous SEPTIN14 coding sequences were obtained from the NCBI Orthologs database [30]. One-to-one orthologous coding sequences across representative mammalian species were downloaded together with their corresponding protein sequences. Multiple sequence alignment was performed using MUSCLE (v5.1) with default parameters on a local Linux environment [36]. The resulting protein alignment was used to generate a codon-based nucleotide alignment while preserving codon structure and reading frame. The alignment corresponding to the 3′ terminal exon was extracted based on exon boundary coordinates defined on the human SEPTIN14 reference annotation.

### Selection pressure analysis

Codon-based selection analyses were performed using site-level models implemented in HyPhy (v2.5) via the Datamonkey web server (https://www.datamonkey.org) [37]. FEL was used to assess purifying (negative) selection acting on the coding sequence [38], and MEME was used to detect episodic deviations from purifying selection [39]. Analyses were conducted separately for the full-length SEPTIN14 coding sequence and for the isolated 3′ terminal exon region, with exon boundaries defined based on the human SEPTIN14 reference annotation. Results were summarized as the dominant mode of selection and the number of sites meeting a reporting threshold of *p* ≤ 0.1.

## Supplementary Information

Supplementary Material 1 [40, 41, 42].

## Data Availability

All data analyzed in this study were obtained from publicly available resources. Genome assemblies, gene annotations, and comparative alignment data were retrieved from the UCSC Genome Browser, Ensembl, and Genomicus. Expression data were obtained from the Genotype-Tissue Expression (GTEx) project. Selection inference was performed externally using Datamonkey (FEL and MEME). All analyses and downstream processing were performed using custom Python scripts. All custom scripts used for block-based locus dispersion analysis, chromosome painting visualization, and alignment-aware integration of selection signals are publicly available at https://github.com/genoevo7/propagation-of-processed-pseudogene-annotation-T2T . Any additional data or materials required to support the findings of this study will be made available by the author upon reasonable request.
