# Supplementary material for: Boundary-associated propagation of a processed pseudogene dissects pre-existing limitations of genome annotation in the T2T era
Source: Mob DNA. 2026 Feb 17;17:9. doi: 10.1186/s13100-026-00394-z (PMC13015009; doi:10.1186/s13100-026-00394-z)
Supplement: Supplementary file 1 — Supplementary Material 1 [40, 41, 42]. [file 13100_2026_394_MOESM1_ESM.pdf]

Supplementary Figure

CICP gene	Locus ID	GRCh38	T2T-CHM13
CICP1	CICP1	chrY:25,384,992-25,391,811	chrY:24,581,421-24,584,241
CICP2	CICP2	chrY:24,274,337-24,281,140	chrY:24,581,436-24,584,241
CICP2	CICP2-1	NA	chrY:26,201,385-26,204,190
CICP3	CICP3	chr1:70,092-706,903	chr1:148,607-151,413
CICP4	CICP4	chr20:64,286,187-64,292,998	chr20:66,110,652-66,113,464
CICP5	CICP5	chr1:223,943,605-223,950,416	chr1:223,137,394-223,140,205
CICP6	CICP6	chr3:198,219,497-198,226,310	chr3:200,955,197-200,958,011
CICP7	CICP7	chr1:465,101-489,906	NA
CICP7	CICP7-1	NA	chr9:150,553,852-150,556,658
CICP8	CICP8	chr7:56,358,458-56,365,270	chr7:56,520,952-56,523,766
CICP9	CICP9	chr10:38,448,987-38,455,365	chr10:38,496,996-38,499,375
CICP10	CICP10	chr2:242,115,856-242,120,053	chr2:242,629,521-242,629,719
CICP11	CICP11	chr7:55,732,779-55,739,605	chr7:55,897,035-55,899,862
CICP12	CICP12	chr7:55,794,034-55,798,876	chr7:55,958,254-55,959,097
CICP13	CICP13	chr1:222,464,094-222,470,882	chr1:221,702,044-221,704,832
CICP14	CICP14	chr7:128,651,962-128,658,791	chr7:129,968,502-129,971,332
CICP15	CICP15	chr5:181,325,049-181,331,839	NA
CICP16	CICP16	chr4:118,631,970-118,638,782	chr4:121,941,271-121,944,084
CICP17	CICP17	chr7:51,378,284-51,384,660	chr7:51,543,546-51,545,906
CICP18	CICP18	chr6:142,230-145,994	chr1:148,603-151,415
CICP18	CICP18-202	NA	chr1:138,270-151,217
CICP19	CICP19	chr19:189,239-195,595	chr19:144,496-146,853
CICP20	CICP20	chr7:45,812,216-45,819,016	chr7:45,977,963-45,980,764
CICP21	CICP21	chr1:243,045,782-243,051,619	NA
CICP22	CICP22	chr7:39,792,593-39,797,911	chr7:39,954,144-39,955,463
CICP23	CICP23	chr11:119,312-126,123	chr11:149,487-152,299
CICP24	CICP24	chr7:63,764,257-63,771,047	chr7:64,976,173-64,978,964
CICP25	CICP25	chr16:90,170,062-90,175,865	NA
CICP26	CICP26	chr1:227,971,390-227,978,193	chr1:227,164,455-227,167,259
CICP27	CICP27	chr1:127,025-134,836	chr16:96,260,210-96,264,022
CICP27	CICP27-1	NA	chr7:61,189-65,009
CICP28	CICP28	chr7:56,801,336-56,808,148	NA

Supplementary Figure 1. Assembly-dependent genomic coordinates of CICP loci in GRCh38 and T2T-CHM13

This data lists the genomic locations of CICP family members in the GRCh38/hg38 and T2T-CHM13 assemblies, highlighting differences in locus resolution between the two references. For each CICP gene, the corresponding chromosomal coordinates are shown for both assemblies where available. Entries labeled with the suffix “-1” or “-202” represent additional loci resolved in the T2T-CHM13 assembly that do not have a direct one-to-one counterpart in GRCh38. The “-1” suffix denotes an additional locus associated with a CICP family member that lacks a corresponding GRCh38 annotation, whereas the “-202” suffix indicates a structurally distinct additional locus resolved within the same CICP family in T2T-CHM13, often in repeat-rich or duplication-prone genomic regions. The suffix “-201” is intentionally not used in this study to avoid ambiguity with existing annotations. Loci marked as “NA” indicate cases where a corresponding locus could not be identified in the respective assembly.

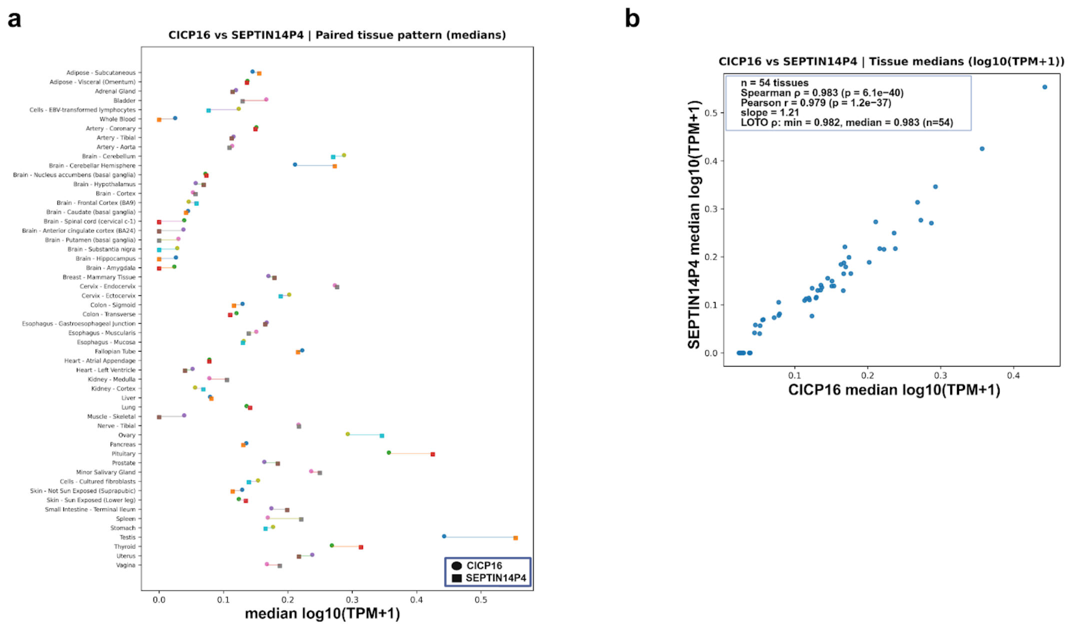

Supplementary Figure 2. Tissue-level co-expression pattern of CICP16 and SEPTIN14P4 pair.

(a) Paired tissue-wise median expression levels of CICP16 and SEPTIN14P4 across 54 GTEx tissues, shown on a log10(TPM+1) scale. For each tissue, the median expression values of the two loci are displayed side by side, enabling direct comparison of their tissue-to-tissue variation. Among the full set of analyzed CICP-SEPTIN14P pairs, this pair represents one of the few cases in which broadly concordant tissue-level expression patterns are observed. (b) Scatter plot of tissue-wise median expression values (log10(TPM+1)) for CICP16 versus SEPTIN14P4 across the same 54 tissues. Each point corresponds to one tissue. Summary statistics, including Spearman's rank correlation, Pearson correlation, linear regression slope, and leave-one-tissue-out (LOTO) robustness metrics, are shown in the inset. These metrics indicate a strong and stable correlation for this specific pair. This figure highlights that coordinated tissue-level expression is not a general property of CICP-SEPTIN14P pairs. Instead, among the examined pairs, only a single representative example shows this pattern. The observed co-expression should therefore not be interpreted as a necessary feature of CICP propagation or annotation, but may represent one of several sufficient conditions under which expression concordance can arise in specific genomic or structural contexts. This example is included for completeness and to illustrate the range of expression relationships observed across pairs, without implying functional coupling or regulatory dependence.

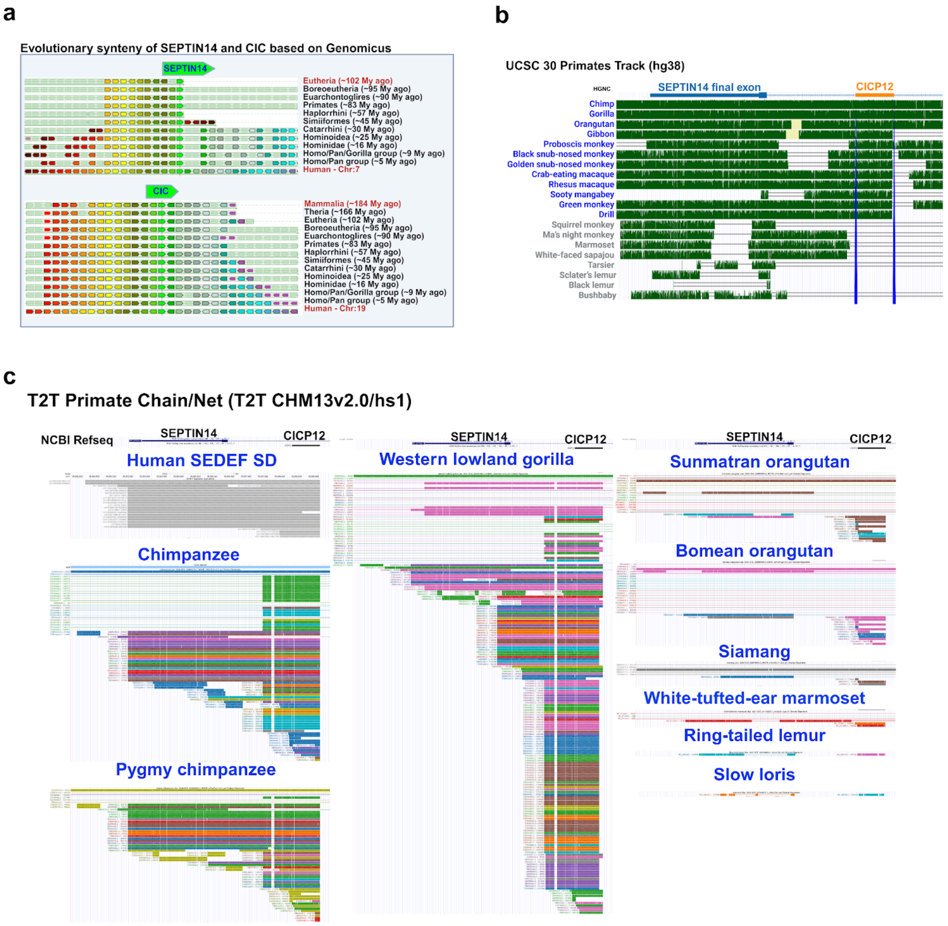

Supplementary Figure 3. Phylogenetic presence of the SEPTIN14-CICP12 locus across primate genomes.

This supplementary figure was included to assess the presence or absence of the SEPTIN14-CICP12 locus across primate lineages, with a particular focus on great apes, rather than to infer the precise timing or mechanism of the original insertion event. (a) Evolutionary synteny of SEPTIN14 and CIC based on Genomicus (Version 110.01), illustrating the conserved genomic context of the parental SEPTIN14 locus and the CIC gene across mammalian and primate lineages. This panel provides a broad evolutionary framework for evaluating lineage-specific retention or absence of the locus, without implying insertion timing. (b) UCSC 30-primate multiple alignment track (hg38), highlighting the genomic region encompassing the SEPTIN14 final exon and the CICP12 locus. The alignment illustrates the presence of the corresponding genomic interval in great apes and selected primates, while gaps or discontinuities reflect absence or unresolved regions in more distant lineages. (c) T2T primate chain/net alignments projected onto the T2T-CHM13v2.0/hs1 reference, showing the structural projection of the SEPTIN14-CICP12 window across great ape and non-great ape primate assemblies. This analysis is intended solely to determine whether a homologous locus is detectable in each lineage, and not to reconstruct the timing, directionality, or mechanism of the original insertion or subsequent structural events.

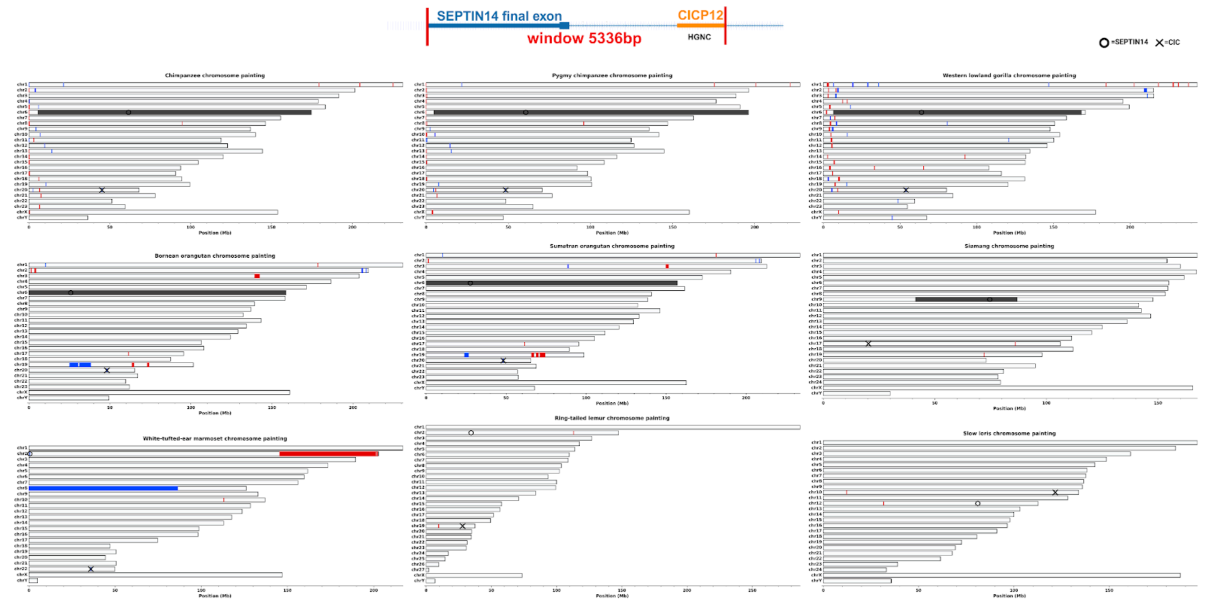

Supplementary Figure 4. Chromosome-level dispersion of the SEPTIN14-CICP12 genomic window across primate genomes.

Chromosome painting representations showing the distribution of chain-mapped alignments corresponding to a 5,336 bp human genomic window encompassing from the SEPTIN14 final exon to the adjacent CICP12 locus (T2T-CHM13v2.0/hs1 reference) across multiple primate assemblies. For each species, horizontal bars represent chromosomes scaled by physical length, with colored ticks indicating chain-mapped alignment blocks derived from the human query window. The plots visualize how a single human locus projects onto one or multiple chromosomes in each genome, reflecting lineage-specific patterns of dispersion and structural fragmentation. This supplementary analysis is intended to document the presence and chromosomal distribution of homologous sequences across primates, with particular emphasis on great apes, rather than to infer the timing, directionality, or molecular mechanism of the original insertion event. The observed differences in dispersion patterns among species highlight variation in structural propagation and genomic context, without implying functional conservation or evolutionary causality.

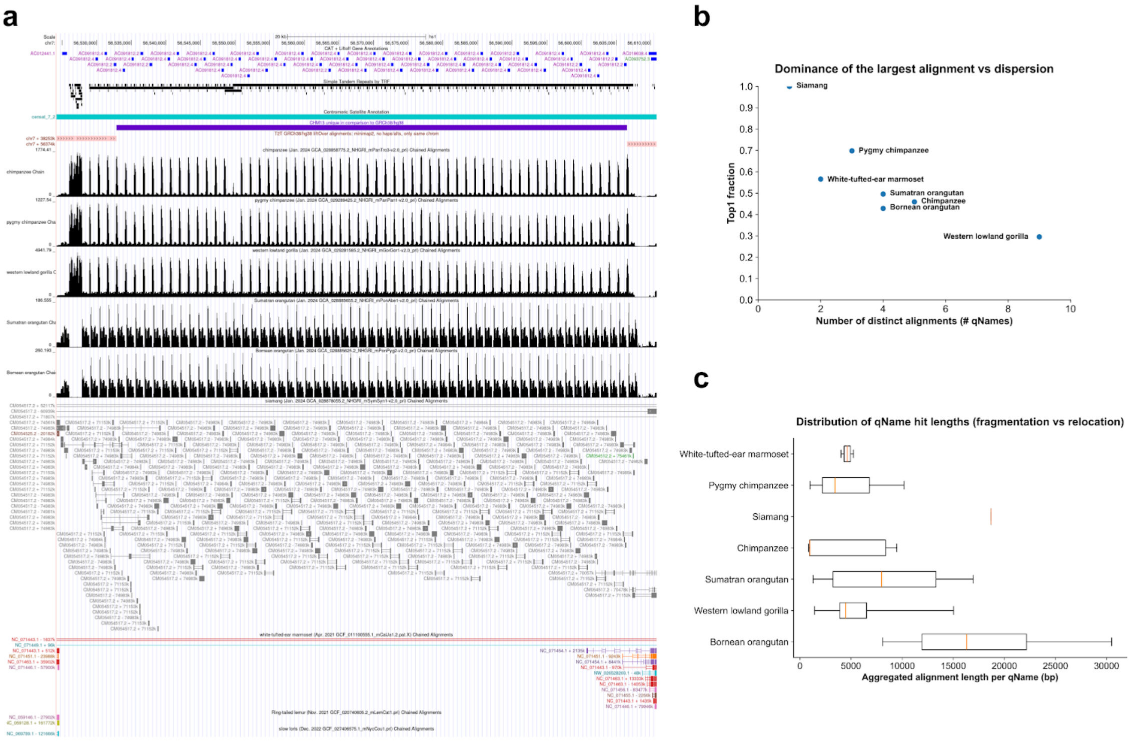

Supplementary Figure 5. Fragmentation and dispersion patterns of chain-mapped alignments for the chr7:56524206-56610877 (T2T unique) genomic window across primate genomes.

(a) Genome browser view of the chr7:56524206-56610877 locus in the T2T-CHM13v2.0/hs1 assembly, showing gene annotations, repeat content, centromeric satellite annotation, and SEDEF-defined segmental duplication tracks. Below, chain-mapped alignments from multiple primate assemblies are displayed as coverage profiles across the same human genomic window, illustrating species-specific patterns of alignment density and fragmentation within a repeat-rich, boundary-associated genomic context resolved by T2T-level assembly. In panels (b) and (c), quantitative summaries were derived from chain-mapped alignments restricted to a single human genomic window (chr7:56524206-56610877, T2T-CHM13v2.0/hs1), enabling cross-species comparison of dispersion and fragmentation patterns. (b) Relationship between the dominance of the largest alignment block (Top1 fraction) and the number of distinct alignment targets (qNames) per species. Each point represents a primate genome, summarizing how the total aligned sequence is distributed across multiple genomic locations. Species with higher qName counts tend to show lower dominance of a single alignment block, consistent with increased structural dispersion. (c) Distribution of aggregated alignment lengths per qName across species. Boxplots summarize the extent to which homologous sequences are concentrated in larger contiguous blocks versus fragmented across multiple shorter segments. These distributions highlight interspecies differences in fragmentation and dispersion of homologous sequence blocks, without implying specific duplication mechanisms or evolutionary directionality. Together, this supplementary figure provides a quantitative and descriptive characterization of how a single human genomic window projects onto multiple primate genomes at

the level of chain-based alignments. The analyses are intended to document structural dispersion and fragmentation patterns across lineages.

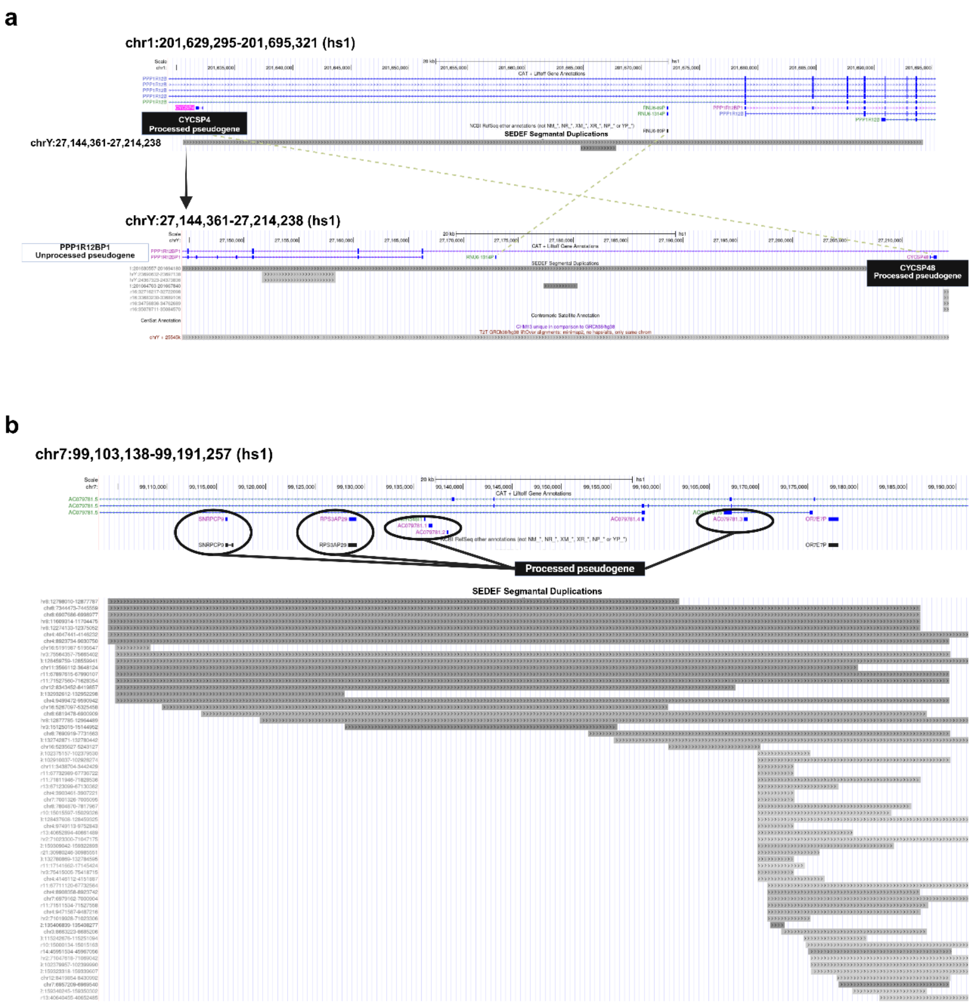

Supplementary Figure 6. Segmental duplication-associated propagation of loci annotated as processed pseudogenes within protein-coding gene contexts

Representative genomic loci illustrating segmental duplication (SD)-associated propagation of sequences annotated as processed pseudogenes and embedded within protein-coding gene contexts. (a) A locus on chr1 containing CYCSP4, annotated as a processed pseudogene, located within the genomic span of the protein-coding gene PPP1R12B, and a corresponding duplicated region on chrY harboring CYCSP4B, also annotated as a processed pseudogene, within an SD block. In the same chrY region, PPP1R12BP1 is annotated as an unprocessed pseudogene. Because these sequences are embedded within coding gene contexts, the original formation mechanism of the source locus, whether arising from RNA-mediated retroposition or from duplication of a protein-coding gene fragment, cannot be unambiguously determined; however, the shared SD boundaries indicate duplication-mediated propagation. (b) An additional locus on chr7 showing multiple annotated processed pseudogenes, including SNRPCP9 and RPS3AP29, co-localized with the protein-coding gene AC079781 (transcripts AC079781.1 and AC079781.2) and the unprocessed pseudogene

OR7E7P, distributed across extensive SD regions. The preservation of protein-coding gene context across duplicated blocks suggests that such loci may, in principle, be amenable to analyses based on synteny conservation and selection pressure, although no such analyses were performed here. These loci are shown for illustrative purposes only and were not included in any quantitative analyses.

Supplementary Methods

Expression analysis for a representative propagated pair

For one representative CICP-SEPTIN14P pair, tissue-wise median expression values were obtained from GTEx (GTEx Analysis V11)[40]. Expression values were transformed as log10(TPM+1). Spearman and Pearson correlations, linear regression slope, and leave-one-tissue-out robustness metrics were computed across tissues in Python (v3.11). This analysis was included as a descriptive example and was not interpreted as a general property of propagated loci.

Synteny analysis using Genomicus

Syntenic relationships surrounding the parental genes CICP and SEPTIN14 were examined using Genomicus (Version 110.01)[41]. Gene-centered synteny views were used to assess conservation and rearrangement of neighboring gene order across primate lineages, with human as the reference. Conservation of the parental genes served as positional anchors to evaluate relative gene order, local syntenic context, and lineage-specific rearrangements in duplication-rich regions. This analysis was used to establish the relative syntenic environment and ordering relevant to locus origin and was interpreted descriptively without assuming direct orthology of duplicated segments.

30-way multiple genome alignment visualization

To visualize cross-species alignment patterns in structurally unstable regions, the UCSC 30-way multiple genome alignment[42] was inspected using the hg38 human reference due to tool and data availability limitations. Given known variability in assembly quality and alignment continuity across species, particularly in duplication- and repeat-rich regions, the 30-way alignment was presented for visual reference only. Alignment patterns were not used for direct evolutionary inference or quantitative interpretation and were excluded from downstream analytical conclusions.

Methodological scope and availability

Primary analytical procedures and computational workflows are described in the main Methods section. Additional methodological details are available from the corresponding author upon reasonable request. References cited in the Supplementary Methods are numbered consecutively with those in the main reference list and are not presented as a separate reference section.
